# Functional significance of germline *EPAS1* variants

**DOI:** 10.1530/ERC-20-0280

**Published:** 2020-12-07

**Authors:** Trisha Dwight, Edward Kim, Karine Bastard, Diana E Benn, Graeme Eisenhofer, Susan Richter, Massimo Mannelli, Elena Rapizzi, Aleksander Prejbisz, Mariola Pęczkowska, Karel Pacak, Roderick Clifton-Bligh

**Affiliations:** 1 Cancer Genetics Laboratory, Kolling Institute, Royal North Shore Hospital, St Leonards, New South Wales, Australia; 2 University of Sydney, Sydney, New South Wales, Australia; 3 Institute of Clinical Chemistry and Laboratory Medicine, University Hospital Carl Gustav Carus, Technische Universität Dresden, Dresden, Germany; 4 Department of Medicine III, University Hospital Carl Gustav Carus, Technische Universität Dresden, Dresden, Germany; 5 Department of Experimental and Clinical Medicine, University of Florence, Florence, Italy; 6 Department of Hypertension, National Institute of Cardiology, Warsaw, Poland; 7 National Institutes of Health, Bethesda, Maryland, USA; 8 Department of Endocrinology, Royal North Shore Hospital, St Leonards, New South Wales, Australia

**Keywords:** pheochromocytoma, paraganglioma, hypoxia- inducible factors, *EPAS1*, HIF-2α, VHL

## Abstract

Mosaic or somatic *EPAS1* mutations are associated with a range of phenotypes including pheochromocytoma and/or paraganglioma (PPGL), polycythemia and somatostatinoma. The pathogenic potential of germline *EPAS1* variants however is not well understood. We report a number of germline *EPAS1* variants occurring in patients with PPGL, including a novel variant c.739C>A (p.Arg247Ser); a previously described variant c.1121T>A (p.Phe374Tyr); several rare variants, c.581A>G (p.His194Arg), c.2353C>A (p.Pro785Thr) and c.2365A>G (p.Ile789Val); a common variant c.2296A>C (p.Thr766Pro). We performed detailed functional studies to understand their pathogenic role in PPGL. In transient transfection studies, *EPAS1*/HIF-2α p.Arg247Ser, p.Phe374Tyr and p.Pro785Thr were all stable in normoxia. In co-immunoprecipitation assays, only the novel variant p.Arg247Ser showed diminished interaction with pVHL. A direct interaction between HIF-2α Arg247 and pVHL was confirmed in structural models. Transactivation was assessed by means of a HRE-containing reporter gene in transiently transfected cells, and significantly higher reporter activity was only observed with *EPAS1*/HIF-2α p.Phe374Tyr and p.Pro785Thr. In conclusion, three germline *EPAS1* variants (c.739C>A (p.Arg247Ser), c.1121T>A (p.Phe374Tyr) and c.2353C>A (p.Pro785Thr)) all have some functional features in common with somatic activating mutations. Our findings suggest that these three germline variants are hypermorphic alleles that may act as modifiers to the expression of PPGLs.

## Introduction

Cells sense and respond to low oxygen conditions in large part *via* hypoxia-inducible factor (HIF)-mediated transcription (Kaelin *et al.* 2016). In normoxia, HIF-α is hydroxylated by oxygen-dependent prolyl hydroxylases (PHDs) then ubiquitinated by pVHL-E3 ligase for proteasomal degradation (Maxwell *et al.* 1999, Hon *et al.* 2002). In hypoxia, PHDs are inactive leading to HIF-α stabilization and transactivation of hypoxia-responsive genes, typically via heterodimers with HIF-β also known as aryl hydrocarbon receptor nuclear translocator (ARNT) (Dengler *et al.* 2014). Two HIF-α isoforms, HIF-1α and HIF-2α have a high sequence homology encoding for two oxygen-dependent degradation domains, as well as two transactivation domains (Erbel *et al.* 2003, Schödel *et al.* 2011). Although they share common target genes, studies have shown that regulation and function of these proteins differ in tumor cells (Loboda *et al.* 2010, Qin *et al.* 2014). Their transcriptional responses include genes associated with angiogenesis, proliferation, anaerobic metabolism and resistance to apoptosis. As these biological consequences align with canonical hallmarks of cancer, numerous studies have reported that tumorigenesis is frequently accompanied by illegitimate activation of hypoxia responses at normal oxygen levels (‘pseudohypoxia’, or the Warburg effect) (Warburg 1956, Ruan *et al.* 2009).

Pheochromocytomas and paragangliomas (PPGLs) are tumors of the adrenal medulla and autonomic ganglia, respectively, that often demonstrate this HIF-dependent pseudohypoxic switch of energy metabolism, in particular when associated with specific hereditary syndromes including *Von Hippel-Lindau (VHL), SDHA, SDHB, SDHC, SDHD, SDHAF2, Egl nine homolog 1 (EGLN1/PHD2), EGLN2(PHD1), malate dehydrogenase 2 (MDH2)*, and *fumarate hydratase (FH)* (Dahia *et al.* 2005, Robledo *et al.* 2017). A direct role for HIF-2α in the pathogenesis of PPGL was first suggested by Zhuang *et al*. in 2012 when somatic gain-of-function variants were identified in patients with PPGL, somatostatinoma and polycythemia. Now termed the Pacak-Zhuang syndrome, these *EPAS1* variants cluster around proline 531, one of two HIF-2α hydroxylation residues. Functional studies have shown that these EPAS1 mutants are not fully hydroxylated by PHD2 and are stable in normoxia (Pacak *et al.* 2013, Yang *et al.* 2013, Jochmanova & Lazurova 2014). These *EPAS1* variants are exclusively somatic or mosaic (Favier *et al.* 2012, Buffet *et al.* 2014, Crona *et al.* 2014, Comino-Méndez *et al.* 2013, Yang *et al.* 2015). There have been few case reports of PPGL- associated with the germline *EPAS1* variant c.1121T>A, p.(Phe374Tyr) (Lorenzo *et al.* 2013, Welander *et al.* 2014, Taïeb *et al.* 2016) but the pathogenicity of that variant remains controversial.

We report the occurrence of several germline *EPAS1* variants in patients with PPGLs, and performed detailed functional studies to validate the clinical significance of this observation. We report three germline *EPAS1* variants (c.739C>A (p.Arg247Ser), c.1121T>A (p.Phe374Tyr) and c.2353C>A (p.Pro785Thr)) all have functional features in common with the well-known activating mutation p.Pro531Thr. Our findings suggest that these three germline variants are hypermorphic alleles that may act as modifiers to the expression of PPGLs.

## Materials and methods

### Patient cohort

DNA was extracted or provided from peripheral blood leukocytes of 300 individuals with PPGL (Australian (*n* = 133), German (*n* = 45), Italian (*n* = 52) and Polish (*n* = 70)). The Australian cohort was sequenced as part of validation for next-generation sequencing, and most samples were de-identified for this purpose without access to corresponding clinical data; two subjects previously enrolled in the Australian SDH Consortium provided written informed consent for use of their de-identified clinical details (Northern Sydney Local Health District Human Research Ethics Committee, Kolling Neuroendocrine Tumour Bank Protocol #11011-361 M and Australian SDH Consortium Protocol #1103-101 M).

### Sequencing

Sequencing was performed as previously described (Dwight *et al.* 2016) using a custom gene panel (TruSeq® Custom Amplicon Assay, Illumina) encompassing the protein-coding and flanking intronic regions of *EPAS1* (NM_001430.5, NP_001421.2). Briefly, DNA libraries were prepared (using 250 ng of DNA from each sample) and sequenced on a MiSeq platform (using 2 × 150 bp paired end reads) according to the manufacturer’s instructions (Illumina). FASTQ files (containing reads and their base call quality scores) were generated for each sample; alignment of reads (banded Smith-Waterman algorithm) and variant calling (GATK (Lin *et al.* 2011)) was processed by MiSeq Reporter (version 2.0, Illumina). Annotation of functional consequences to variant calls was performed using ANNOVAR (version 2013 Jul (Liu *et al.* 2007)), which incorporates various *in silico* tools, including (but not limited to) PolyPhen-2, SIFT, MutationTaster. Visualization of reads was performed using IGV (v2.1). All *EPAS1* variants were confirmed on Sanger sequencing.

### Penetrance and confidence interval calculation

Bayesian calculation of the conditional probability of disease (penetrance) given the genotype was performed using methods as previously described (Minikel *et al*. 2016, Benn *et al*. 2018).

### Plasmid and site-directed mutagenesis

Plasmid pCMV6-EPAS1-GFP (RG208604, Origene) was used as the template for site-directed mutagenesis (QuikChange Lightning Site-Directed Mutagenesis Kit, Agilent) to produce *EPAS1* variants c.581A>G (p.His194Arg), c.739C>A (p.Arg247Ser), c.1121T>A (p.Phe374Tyr), c.2296A>C (p.Thr766Pro), c.2353C>A (p.Pro785Thr) and c.2365A>G (p.Ile789Val). Additionally, *EPAS1* c.1591C>A (p.Pro531Thr) known to affect a critical hydroxylation site of HIF-2α and previously associated with PGL (Toledo *et al*. 2013) was generated as a positive control. Plasmids were sequenced to confirm the presence of WT or variant sequences and confirm an in-frame GFP tag. Commercially available renilla luciferase reporter for *vascular endothelial growth factor A (VEGFA)* promoter – associated with hypoxia signaling – was obtained (#S722028, SwitchGearGenomics, CA, USA).

### Cell culture and transfection

HEK293 and HeLa cells were both cultured in DMEM (Life Technologies) with 10% fetal bovine serum (FBS). PC12 cells required RPMI (Life Technologies) supplemented with 5% FBS and 10% heat-inactivated horse serum (Life Technologies). Experiments involving plasmid transfection began with seeding of 1 × 10^5^ cells/well for HEK293 and HeLa cells, whereas PC12 cells were plated at 3 × 10^5^ cells/well in six-well plate 24 h prior to 2 µg/well of plasmid DNA transfection experiments using OptiMEM (Life Technologies) and Lipofectamine 2000 (Life Technologies). Immediately upon transfection of plasmid DNA, designated 6-well plates were exposed to low-oxygen environment using hypoxia chamber XVIVO system (BioSpherix, NY, USA) which was used to incubate cells in 1% O_2_ at 37°C for 48 h. The level of O_2_, N_2_, CO_2_ were logged in real-time using Oxycycler Watview software (BioSpherix) to ensure appropriate conditions were maintained.

### Stability of HIF-2α variants

Whole cell lysates were prepared using RIPA buffer and quantified to assess stability of transfected GFP-tagged WT HIF-2α under normoxic conditions (21% O_2_), compared with hypoxic conditions (1% O_2_) as a positive control. Stability of GFP-tagged HIF-2α variants was assessed using cells conditioned under normoxic conditions. Extracts were mixed with NuPAGE® LDS sample buffer (Invitrogen) and dithiothreitol and incubated at 95°C for 5 min and separated by SDS-PAGE (4–12% NuPAGE Bis-Tris gels, Invitrogen) under reducing conditions. Proteins were transferred (nitrocellulose membrane) and the membrane blocked with 5% skim milk (in TBST) for 1 h at room temperature. The membranes were probed with the following antibodies: GFP (dilution 1:2000, Roche (11814460001)), and GAPDH (dilution 1:5000, Cell Signaling (D16H11)) and incubated overnight at 4°C. Immunoblots were washed three times with TBST for 5–10 min and incubated with the relevant secondary antibody conjugated to horseradish peroxidase (HRP). Blots were then washed (three times in TBST for 5 min) and protein detected (ECL Plus Western Blotting Detection Reagent (GE Healthcare)) on a LAS-3000 (Fujifilm, Brookvale, Australia).

### Co-immunoprecipitation

To determine whether mutant HIF-2α disrupts the normal interaction of HIF-2α with pVHL or HIF-1β, GFP-tagged WT or mutant HIF-2α were blotted after endogenous pVHL or HIF-1β mediated pulldown. Briefly, 48 h post-transfection, cells were washed (PBS), pelleted and lysed using co-immunoprecipitation (Co-IP) buffer (20 mM Tris pH 7.5, 150 mM NaCl, 1 mM EGTA, 1 mM EDTA, 0.1% Triton X100). Dynabeads^®^ M-280 sheep anti-rabbit IgG (#026102, Life Technologies) were incubated with either rabbit IgG antibody (dilution 1:2000, #31460, Thermo-Fisher) for negative control, pVHL polyclonal rabbit antibody (dilution 1:2000, #2738, Cell Signaling Technology) or HIF-1β polyclonal rabbit antibody (dilution 1:2000, #5537, Cell Signaling Technology) for 2 h prior to washing; then incubated overnight with cell extracts at 4°C under gentle rotation. Proteins not associated with pVHL or HIF-1β were removed (3 × 10 min gentle agitation washes) using Co-IP lysis buffer with a higher salt concentration (500 mM NaCl). To remove immunoprecipitated material from beads, cell lysates were mixed with NuPAGE® LDS sample buffer (Invitrogen) and dithiothreitol and incubated at 95°C for 5 min. Extracts were removed from beads and separated by SDS-PAGE (4–12% NuPAGE Bis-Tris gels, Invitrogen) under reducing conditions. Proteins were transferred, blocked, probed and protein detected as outlined previously.

### Cyclohexamide treatment

To assess the half-life of GFP-tagged WT or mutant HIF-2α proteins in plasmid transfected HEK293 cells, 100 µg/mL of cycloheximide (CHX; #C0934, Sigma) was added to inhibit protein synthesis at 60, 30 and 10 min prior to protein extraction.

### Structural modeling

To assess the interaction between pVHL and *EPAS1* residue p.Arg247, the structure of human HIF-2α was extracted from the crystal structure of HIF-2α heterodimerized with ARNT-C-terminal PAS domain (PDB code: 3F1P, chain A) (Scheuermann *et al.* 2009). The engineered Glu247 found in HIF-2α crystal structure was changed back to Arg247. The structure covers the PAS-B domain (residues 236 from 349). Thus, a homology model for the bHLH and PAS-A domains of HIF-2α (residues 27–235) was built using the crystal structure of mouse Hif-2α, extracted from the heterodimeric complex HIF-2α-ARNT crystal structure (PDB code: 4ZP4, chain B) (Wu *et al*. 2015). The sequence identity between mouse Hif-2α and human HIF-2α is 98% over the bHLH-PAS domains. The structure of pVHL was extracted from the crystal structure of hydroxylated HIF-2α peptide (residues 523 to 541) bound to the pVHL/elongin-C/elongin-B complex (PDB code 6BVB; chain V) (Tarade *et al.* 2018). The docking server ClusPro was used to dock pVHL (defined as the ligand) on HIF-2α (defined as a receptor). 101 models were generated. Models for which an interface was predicted between pVHL and HIF-2α that include residues on pVHL known to be in interaction with elongin-C in pVHL/elongin-C/elongin-B complex were excluded. These residues are 79–81, 132, 152–166, 169, 178–184, and 188 on pVHL. Models for which an interface was predicted between pVHL and HIF-2α that include residues on HIF-2α known to be in interaction with DNA or ARNT in the crystal structure of DNA-bound HIF-2α-ARNT structures were excluded from the set of models. These residues are 73, 76, 79, 80–97, 100–108 and 112 on HIF-2α. Finally, three models were remaining. The structures and interaction of three models have been optimized using energy minimization with Maestro software (Schrödinger, D.E. Shaw Research, NY). The lowest energy model has been used for our structural analysis. The dimer elongin C-elongin B was positioned on pVHL according to the crystal structure pVHL/elongin-C/elongin-B (PDB code 6BVB; chain V) (Tarade *et al.* 2018). Images were created with Pymol.

### Luciferase assay

Impact of germline *EPAS1* variants on transcriptional activity of mutant HIF-2α relative to WT was assessed. Promoter activity of *VEGFA* was selected as a downstream transcription target of HIF-2α (Maxwell *et al.* 1999, Meneses & Wielockx 2016). Along with the WT or variant GFP-tagged *EPAS1* plasmids, 10 ng of pLightSwitch_*VEGFA* and 400 ng of pGL3-Basic were also added into the transfection mixture. After 48 h of incubation, passive lysis buffer was used to harvest cells for luciferase assay.

### RNA extraction and qRT PCR

Gene expression analyses were performed to examine whether stabilization of HIF-2α induced hypoxia-responsive genes including cyclin D1 coding *CCND1* (encoding cyclin D1) and *SLC2A1* (encoding glucose transporter 1). Taqman gene expression assays (Thermo-Fisher) were used perform qRT-PCR using probes specific to *CCND1* (#Hs00765553_m1), *SLC2A1* (Hs00892681_m1) and *ACTB* (#Hs99999903_m1, beta actin, endogenous control).

### Statistical analyses

Minor allele frequencies of *EPAS1* variants in our cases and gnomAD (non-cancer) European non-Finnish controls were compared using the Fisher exact test. HIF-2α variant stability in normoxia was measured by densitometry of Western blots, corrected for GAPDH in each case and expressed relative to the value of WT HIF-2α under hypoxia as mean ± s.d. of *n* = 3 experiments. HIF-2α variant interaction with pVHL by co-immunoprecipitation was also measured by densitometry of Western blots, corrected for pVHL and expressed relative to the value of WT HIF-2α under normoxia as mean ± s.d. of *n* = 3 experiments. Differences in protein measurements were analyzed by Student’s t-test using GraphPad Prism version 7.02.

## Results

### Incidental identification of germline *EPAS1* variants

During the validation of a multigene panel for next generation sequencing (NGS), we found six Australian PPGL patients who had *EPAS1* germline variants (c.581A>G (p.His194Arg), c.739C>A (p.Arg247Ser), c.1121T>A (p.Ph374Tyr), or c.2296A>C (p.Thr766Pro)) occurring together with previously identified PPGL-associated germline mutations in other driver genes (Table 1). Notably, five of six of these patients had germline *SDHB* mutations. For two cases annotated clinical data were available. Case #1 (*EPAS1* variant c.581A>G, p.His194Arg) together with *VHL* mutation c.350G>C, p.Trp117Ser) was a man who had resection of a right phaeochromocytoma aged 30 years, and who also had bilateral renal masses, a pancreatic neuroendocrine tumor and recurrent hemangioblastomas consistent with diagnosis of VHL syndrome; his most recent hematocrit values have ranged between 0.45 and 0.52 (normal range 0.39–0.54). Case #3 (*EPAS1* variant c.1121T>A, p.Phe374Tyr together with *SDHB* mutation c.286G>A, p.Gly96Ser) was a woman who had resection of a carotid body paraganglioma at age 52 years, without recurrence 15 years later; her most recent hematocrit values have ranged between 0.47 and 0.49 (normal range 0.35–0.47). It was not possible to verify familial transmission of the *EPAS1* variant in either case.

**Table 1:** Germline *EPAS1* variants in individuals with previously identified PPGL-associated germline drivers.

Patient #	Features	Germline driver gene variant	Germline *EPAS1* variant	
			Nucleotide^a^ change	Amino acid^b^ change
1	PC	*VHL* p.Trp117Ser	c.581A>G	p.His194Arg
2	PGL	*SDHB* c.72+1G>T	c.739C>A	p.Arg247Ser
			c.1121T>A	p.Phe374Tyr
3	PC	*SDHB* p.Gly96Ser	c.1121T>A	p.Phe374Tyr
4	PGL	*SDHB* p.Ile127Ser	c.1121T>A	p.Phe374Tyr
5	PC^met^	*SDHB* p.Gln24*	c.2296A>C	p.Thr766Pro
6	PGL	*SDHB* p.Pro197Arg	c.2296A>C	p.Thr766Pro

^a^NCBI Reference Sequence: NM_001430.5; ^b^NCBI Reference Sequence: NP_001421.2.

met, known to have metastatic disease; PC, pheochromocytoma; PGL, paraganglioma.

On the basis of our preliminary findings that germline *EPAS1* variants may occur together with other germline PPGL predisposition alleles, we extended our *EPAS1* sequence analysis to a larger cohort of 300 individuals with PPGL (Australian (*n* = 133, includes Table 1 subjects), German (*n* = 45), Italian (*n* = 52) and Polish (*n* = 70) subsets; Table 2 and Supplementary Tables 1, 2, see section on supplementary materials given at the end of this article). As shown in Table 2, an additional 15 subjects (of whom five also had germline *SDHx* mutations) were found with germline *EPAS1* alleles: one with *EPAS1* c.830C>T (p.Ala277Val), two with c.1121T>A (p.Ph374Tyr), one with c.1963G>A (p.Gly655Arg), ten with c.2296A>C (p.Thr766Pro) two of whom (#19, 20) also carried c.2353C>A (p.Pro785Thr), and one with c.2365A>G (p.Ile789Val). Combining both cohorts, germline *EPAS1* variants were found no more often in subjects with an *SDHB* mutation (six of 75 subjects), compared with subjects without an *SDHB* mutation (15 of 225, *P = *0.7); nor was prevalence of germline *EPAS1* variants different in those with germline mutations in *VHL*/*SDHx* genes (11 of 176 subjects) compared with others (10 of 124 subjects).

**Table 2:** Germline *EPAS1* variants in validation cohort.

Patient #	Germline driver gene variant	Germline *EPAS1* variant	
		Nucleotide^a^ change	Amino acid^b^ change
7	SDHB	c.830C>T	p.Ala277Val
8	SDHC	c.1121T>A	p.Phe374Tyr
9	SDHD	c.2296A>C	p.Thr766Pro
10	SDHD	c.2296A>C	p.Thr766Pro
11	SDHD	c.2296A>C	p.Thr766Pro
12	None known	c.1121T>A	p.Phe374Tyr
13	None known	c.1963G>A	p.Gly655Arg
14	None known	c.2296A>C	p.Thr766Pro
15	None known	c.2296A>C	p.Thr766Pro
16	None known	c.2296A>C	p.Thr766Pro
17	None known	c.2296A>C	p.Thr766Pro
18	None known	c.2296A>C	p.Thr766Pro
19	None known	c.2296A>C	p.Thr766Pro
		c.2353C>A	p.Pro785Thr
20	None known	c.2296A>C	p.Thr766Pro
		c.2353C>A	p.Pro785Thr
21	None known	c.2365A>G	p.Ile789Val

^a^NCBI Reference Sequence: NM_001430.5; ^b^NCBI Reference Sequence: NP_001421.2.

Using *in silico* tools (Table 3), *EPAS1* variant c.739C>A (p.Arg247Ser) was consistently predicted to be pathogenic, whereas c.581A>G (p.His194Arg), c.830C>T (p.Ala277Val) and c.1121T>A (p.Phe374Tyr) substitutions were predicted to be pathogenic by some but not all tools. *EPAS1* variants c.1963G>A (p.Gly655Arg), c.2296A>C (p.Thr766Pro), c.2353C>A (p.Pro785Thr) and c.2365A>G (p.Ile789Val) were predicted to be benign by all prediction tools.

**Table 3:** *EPAS1* variant characteristics.

*EPAS1*/HIF-2α** variant	MAF^a^ in combined cohort^b^	MAF in gnomAD^c^		*In silico* tools		
		All	European non-Finnish	SIFT^d^	PolyPhen-2^e^	Mutation Taster^f^
p.His194Arg	0.0018**	0.00007	0	D	PD	DC
p.Arg247Ser	0.0017	np	np	D	PD	DC
p.Ala277Val	0.0017*	0.00002	0.00003	T	B	DC
p.Phe374Tyr	0.0087	0.0039	0.0059	T	B	DC
p.Gly655Arg	0.0017	0.0002	0.0004	T	B	P
p.Thr766Pro	0.0206	0.06	0.0166	T	B	P
p.Pro785Thr	0.0034*	0.014	0.0005	T	B	P
p.Ile789Val	0.0017**	0.00002	0.00003	T	B	P

Statistical comparison between *EPAS1* variant MAF in PPGL cases and gnomAD European-non-Finnish controls *P < 0.05; ** *P* < 0.001.

^a^MAF, minor allele frequency (number of cases with allelic variant/total number of alleles); ^b^combined cohort of PPGL cases from Australia, Germany, Italy, and Poland; ^c^gnomAD v.2.1 (non-cancer); ^d^ Vaser * et al*. (2016) SIFT missense predictions for genomes; ^e^Adzhubei * et al*. (2010) A method and server for predicting damaging missense mutations ; ^f^Schwarz * et al*. (2014) Mutation Taster2: mutation prediction for the deep-sequencing age.

B, benign; D, deleterious; DC, disease causing; Np, not present; P, polymorphism; PD, possibly deleterious; T, tolerate.

We then compared allelic frequencies of these variants in PPGL patients with those from the genome aggregation database samples from individuals who were not ascertained for having cancer in a cancer study (gnomAD v2.1.1 non-cancer; Table 3) (Karczewski *et al*. 2020). We note *EPAS1* variants c.2296A>C and c.2353C>A had allelic frequencies in gnomAD of >1%, consistent with being common polymorphisms. Moreover, since there is significant ethnic variation of many of these *EPAS1* variants in non-cancer gnomAD (Supplementary Table 3), we confined statistical comparison to our PPGL cases against European non-Finnish controls (i.e. the relevant ethnic background for our cases). As shown in Table 3, c.581A>G (p.His194Arg), c.830C>T (p.Ala277Val), c.2353C>A (p.Pro785Thr) and c.2365A>G (p.Ile789Val) were more common in PPGL cases than controls. *EPAS1* c.739C>A (p.Arg247Ser) was not seen in gnomAD, although two other rare variants are noted at this codon (c.739C>T (p.Arg247Cys) MAF 8 × 10^−6^; and c.740G>A (p.Arg247His) MAF 8.7 × 10^−5^). Although *EPAS1* variant c.2296A>C (p.Thr766Pro) has uncertain annotation in gnomAD (i.e. it is deemed to be a multi-nucleotide variant), we confirmed this variant by Sanger sequencing in each PPGL case.

### Estimated penetrance of *EPAS1*variants

We next applied Bayesian principles (Minikel *et al.* 2016) that we previously used for *SDHx* variants (Benn *et al.* 2018), to estimate the lifetime penetrance of PPGL for *EPAS1* variants c.1121T>A (p.Phe374Tyr) and c.2353C>A (p.Pro785Thr), taking into account allelic frequencies in our cases vs European non-Finnish gnomAD controls, and assuming estimated population PPGL prevalence of 1/3000 (Benn *et al*. 2018). Using these calculations, c.2353C>A (p.Pro785Thr) has a modest estimated lifetime penetrance for PPGL of 0.22% (CI 0.03–1.13%) whereas c.1121T>A (p.Phe374Tyr) has an estimated lifetime penetrance of only 0.05% (CI 0.02–0.13%, i.e. not significantly different from population prevalence).

### Hypoxia stabilizes WT HIF-2α protein compared to normoxia

**Figure 1:**
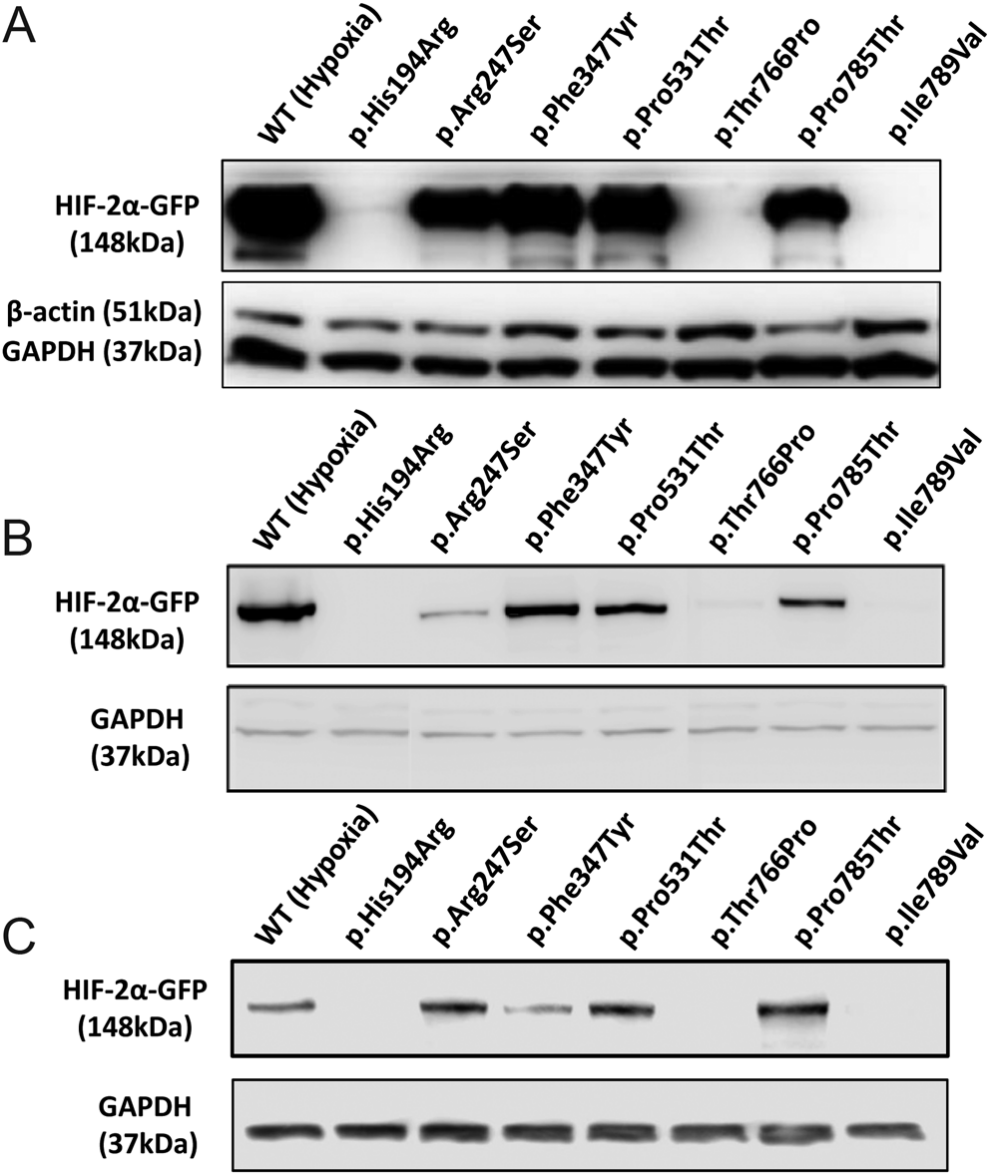
Stability of HIF-2α variants in normoxia. (A) Representative Western blots of lysates from HEK293 cells, transfected with *EPAS1* variants and cultured in normoxia, indicated robust expression of GFP-tagged HIF-2α mutants c.739C>A (p.Arg247Ser), c.1121T>A (p.Phe374Tyr) and c.2353C>A (p.Pro785Thr) relative to pathogenic control p.Pro531Thr, as well as WT HIF-2α in lysates from cells cultured in hypoxia. Endogenous housekeeper proteins B-actin and GAPDH are shown subsequently. Similar stability of HIF-2α mutants were observed in PC12 (B) and HeLa (C) cells, albeit expression level of c.1121T>A (p.Phe374Tyr) was lower for the latter.

To examine the stability of the identified *EPAS1* variants in normoxia, we generated GFP-tagged expression plasmids for each variant (excepting the predicted benign variants p.Ala277Val and p.Gly655Arg) and transfected them into HEK293, PC12 and HeLa cells. We included two positive controls in each experiment: (i) the somatic gain-of-function *EPAS1* mutant p.Pro531Thr in normoxia and (ii) WT HIF-2α-GFP transfected in cells cultured under hypoxic (1%) conditions. We validated that WT-HIF-2α was sensitive to oxygen concentrations, since it was undetectable in transfected cells maintained in normoxia and progressively increased in cells maintained at 5 or 1% oxygen, respectively, (Supplementary Fig. 1). As shown by representative Western blots in Fig. 1, HIF-2α mutants p.Arg247Ser, p.Phe374Tyr and p.Pro785Thr were stable in normoxia in all cellular backgrounds, HEK293 (A), PC12 (B) and HeLa cells (C). When measured by densitometry, levels of these three mutant proteins were not significantly different from p.Pro531Thr or hypoxic WT HIF-2α in HEK293 and HeLa cells, whereas p.Arg247Ser and p.Pro785Thr were mildly lower than controls in PC12 cells (Supplementary Fig. 2). In contrast, HIF-2α mutants p.His194Arg, p.Thr766Pro and p.Ile789Val were not stable in normoxia in any cellular background (Fig. 1 and Supplementary Fig. 2).

**Figure 2:**
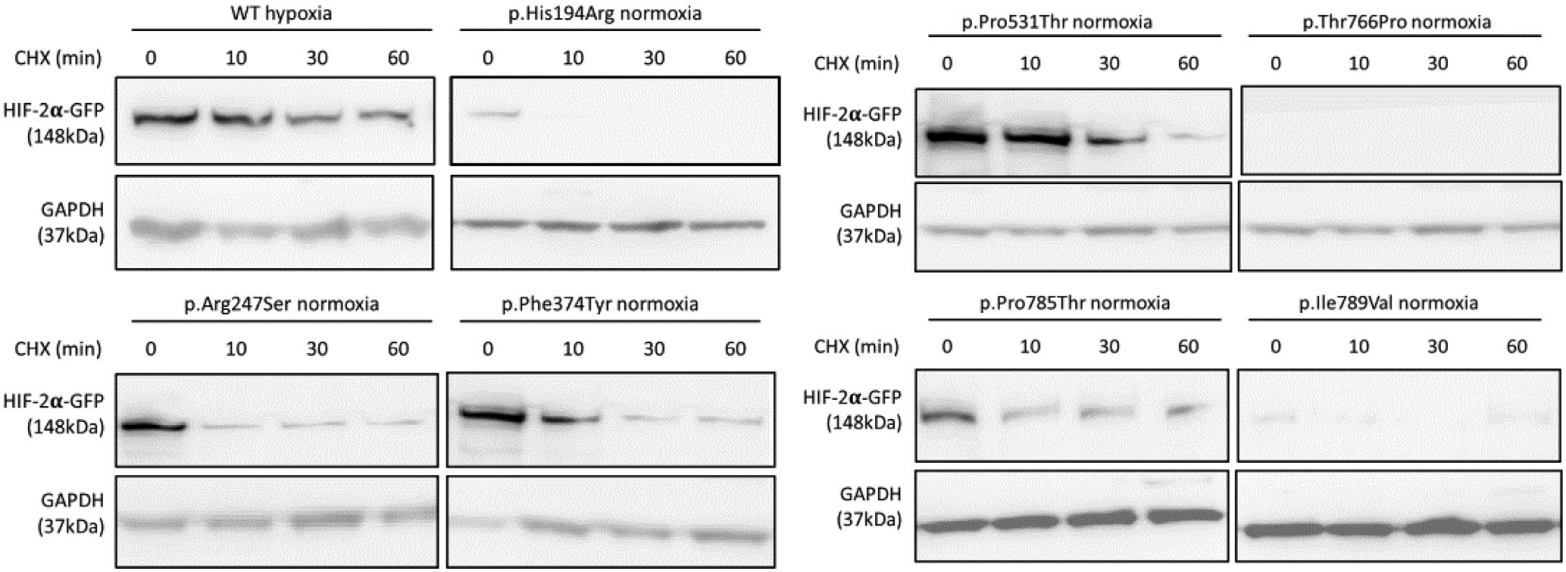
Stability of HIF-2α variants following cycloheximide. Cells exposed to cycloheximide (CHX) over periods of time indicated prolonged stability of WT HIF-2α in hypoxia, and HIF-2α variants c.739C>A (p.Arg247Ser), c.1121T>A (p.Phe374Tyr), p.Pro531Thr, c.2353C>A (p.Pro785Thr) in normoxia.

Transfected HEK293 cells were treated with cyclohexamide to confirm that these HIF-2α levels are not explained by *de novo* protein synthesis. As shown in Fig. 2, HIF-2α variants p.His194Arg, p.Thr766Pro and p.Ile789Val were all rapidly degraded in normoxia 60 min after cycloheximide treatment. In contrast, HIF-2α variants p.Arg247Ser, p.Phe374Tyr and p.Pro785Thr were stable over the course of cycloheximide treatment, similar to the positive control p.Pro531Thr; the stability of WT HIF-2α under hypoxia is shown for reference.

### Interaction between HIF-2α mutants and pVHL is retained except for variant p.Arg247Ser

**Figure 3:**
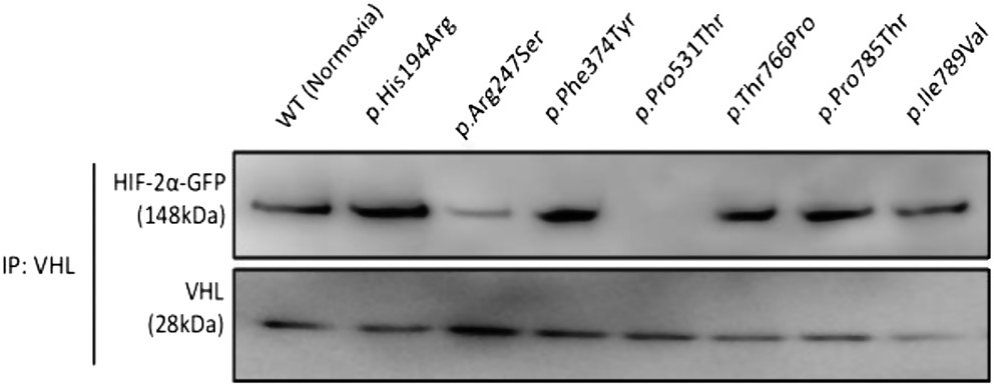
Interaction of HIF-2α variants with pVHL. Representative Western blot following co-immunoprecipitation of GFP-tagged HIF-2α by anti-pVHL antibody. HEK293 cells transfected with WT HIF-2α and cultured in normoxia (48 h) were used as a positive control. The negative control was HIF-2α variant p.Pro531Thr. Reduced HIF-2α-pVHL interaction was only observed for HIF-2α variant c.739C>A (p.Arg247Ser).

Under normoxic condition, HIF-2α is regulated through pVHL mediated degradation. We hypothesized that stability of p.Arg247Ser, p.Phe374Tyr and p.Pro785Thr HIF-2α mutants in normoxia may be due to disrupted interaction between pVHL and HIF-2α. This was assessed by co-immunoprecipitation of HIF-2α mutants from HEK293 cells using magnetic beads coupled to anti-pVHL antibodies. WT HIF-2α extracted from normoxic cells was used as a positive control (Fig. 3 and Supplementary Fig. 3). As expected, gain-of-function positive control p.Pro531Thr HIF-2α mutant did not associate with pVHL in normoxia, due to absence of the prolyl hydroxylation site at residue 531 of HIF-2α (Fig. 3). The novel variant p.Arg247Ser also showed diminished interaction with pVHL. Interestingly, p.Phe374Tyr and p.Pro785Thr HIF-2α mutants that were stable in normoxia retained their interaction with pVHL (Fig. 3). The other HIF-2α mutants p.His194Arg, p.Thr766Pro and p.Ile789Val also retained interaction with pVHL.

### Structural modeling identifies a potential interface between EPAS1 p.Arg247 and pVHL

**Figure 4:**
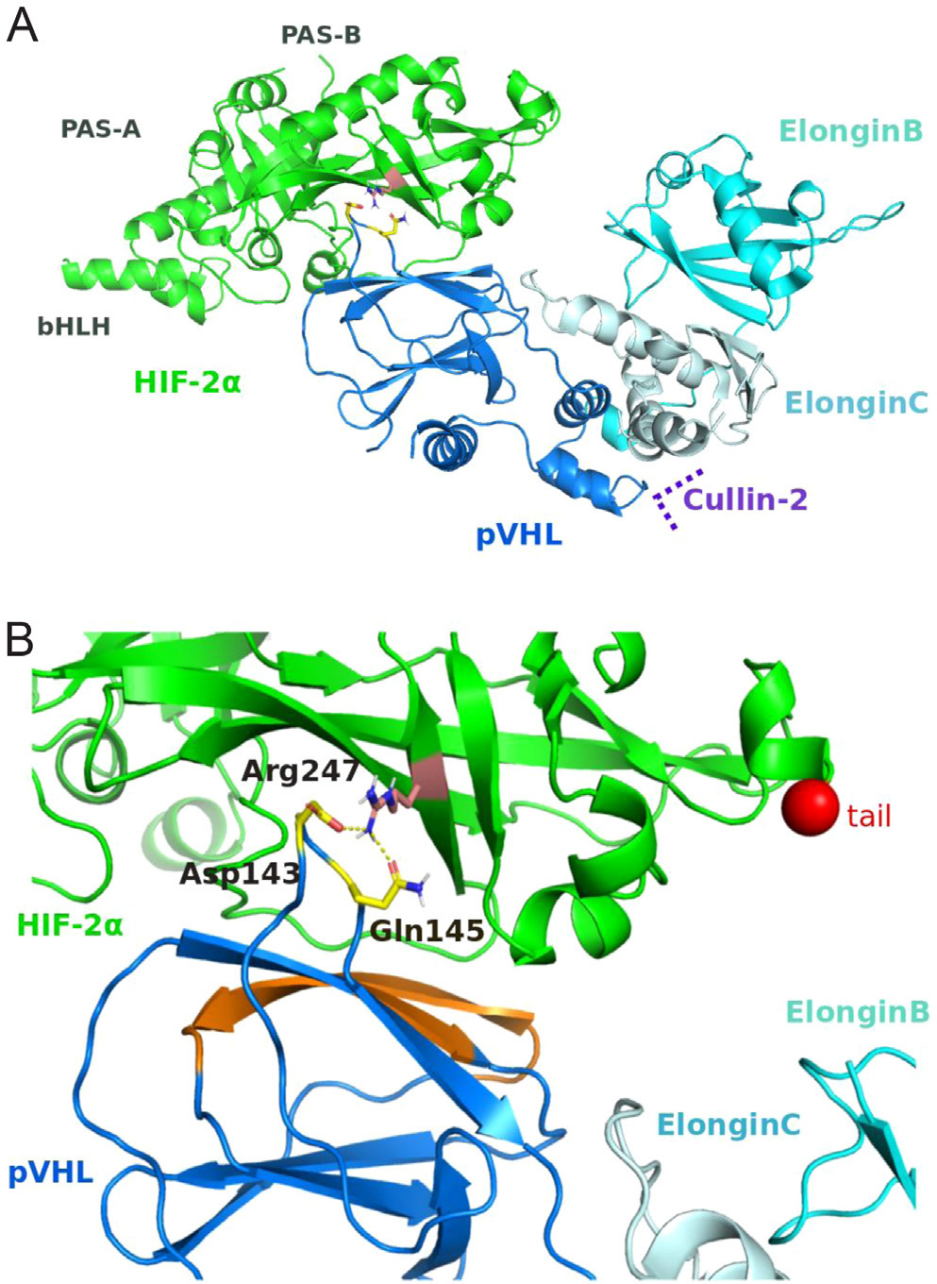
Model of association between HIF-2α and pVHL. (A) Global view of the modeled heteromeric complex HIF-2-pVHL-ElonginC-ElonginB in cartoon representation. For information, the position of the Cullin-2 is indicated according to the crystal structure of Cul2-Rbx1-EloBC-VHL ubiquitin ligase complex (PDB code: 5N4W). Arg247 of HIF-2α, Gln145 and Asp143 of pVHL are represented in stick. (B) Detailed view of the specific interactions between HIF-2α and pVHL. The yellow dash lines represent the electrostatic interactions. The orange on pVHL shows the interfacial residues that interact with HIF-2α peptide (523–541) in the crystal structure of the heteromeric HIF-2α/pVHL/elongin-C/elongin-B complex (PDB code: 6BVB) (Tarade *et al.* 2018). The red sphere indicates the last element of the structure of HIF-2α (residue 360) that has been experimentally determined and modeled in our study (there are no structural data available for the segment 361–522).

We investigated the possible disrupted interaction between pVHL and HIF-2α for mutant p.Arg247Ser. A crystal structure is available for the heteromultimeric complex pVHL/elongin-C/elongin-B in interaction with the 523–540 fragment of HIF-2α (Tarade *et al.* 2018). We also examined other possible binding modes between pVHL and HIF-2α by *in silico* building the complex formed by these two proteins. The best model includes an interface between the L7 loop of the pVHL β-domain and the PAS-B domain of HIF-2α (Fig. 4A). This predicted binding site for HIF-2α on pVHL is about 15 Å away from the HIF-2α binding site described in the crystal structure pVHL/elongin-C/elongin-B/HIF-2α (Tarade *et al.* 2018). We found that Arg247 mediates two critical interactions between HIF-2α and pVHL: a salt bridge with Asp143 and a hydrogen bond with Gln145 on pVHL (Fig. 4B).

### Interaction between HIF-2α mutants and ARNT is unaltered

Cellular hypoxic response requires heterodimerization of HIF-α with ARNT (HIF-β) to act as a transcription factor for activation of various genes. Due to the lack of functional studies of HIF-2α and ARNT pairing in context of PPGL, it was unclear whether our *EPAS1* variants affected this interaction. To investigate this in normoxia, GFP-tagged WT or mutant HIF-2α proteins were overexpressed by tripling transfection input to 6 μg/1 × 10^5^ HEK293 cells to ensure adequate amounts of proteins. Then, co-immunoprecipitation was performed using anti-ARNT on magnetic beads. Subsequent Western blotting analyses showed no obvious difference between various HIF-2α mutants (Supplementary Fig. 4). This indicated that HIF-2α-ARNT interaction was not altered by these *EPAS1* variants.

### HIF-2α induced transcription

**Figure 5:**
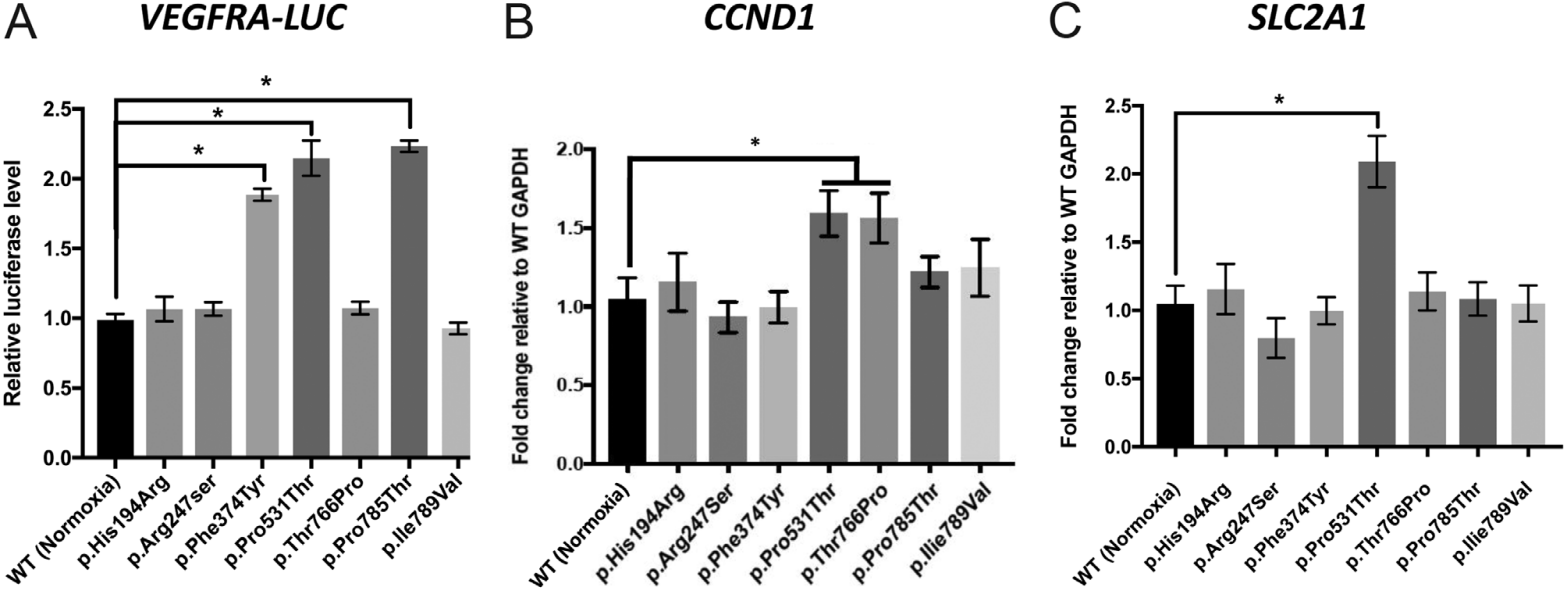
Transcriptional regulation by HIF-2α variants. (A) VEGFA-LUC was transfected in HEK293 cells together with WT or HIF-2α variants as shown, and luciferase was measured in cell lysates after 48 h. Compared to WT HIF-2α, significant transduction of VEGFA-LUC was observed in samples with p. HIF-2α variants Phe374Tyr and c.2353C>A (p.Pro785Thr) alongside p.Pro531Thr that served as positive control (*n* = 3, *P < 0.05). (B) Gene expression assay performed on samples from HEK293 cells that were induced to express WT or mutant HIF-2α in normoxia over 48 h. (A) Compared to WT HIF-2α *CCND1* expression was significantly induced after transfection of somatic gain-of-function p.Pro531Thr HIF-2α mutant (positive control) or our novel c.2296A>C (p.Thr766Pro) variant (*n* = 3, *P < 0.05). (B) Significant induction of *SLC2A1* mRNA was also observed in p.Pro531Thr HIF-2α expressing cells (*n* = 3, *P < 0.05), although** no significant changes were observed in our selected *EPAS1* variants (*n* = 3, *P* > 0.05).

Transactivation by HIF-2α mutants was firstly assessed by means of a HRE-containing reporter gene. HEK293 cells were transfected with WT or mutant *EPAS1* vectors together with pLightSwitch VEGFA, containing the *VEGFA* promoter upstream of the renilla reporter. Significantly higher reporter activity was observed in lysates from cells transfected with *EPAS1/*HIF-2α p.Phe374Tyr, p.Pro785Thr and positive control p.Pro531Thr mutant (Fig. 5A). These results somewhat reflected the effect of HIF-2α mutants on protein stability in normoxia, with the exception of p.Arg247Ser which was not associated with increased reporter transactivation despite apparent protein stability.

To further assess the consequences of stable HIF-2α in normoxia, changes in expression of the genes associated with pseudohypoxia were quantified. This involved extracting total RNA from HEK293 cells transiently transfected with GFP-tagged WT or mutant HIF-2α over 48 h and performing RT for cDNA conversion, followed by quantitative real-time PCR. Two selected genes were; *cyclin D1 (CCND1)* and *solute carrier family 2 member 1 (SLC2A1)* encoding for the glucose transporter protein type 1 (GLUT1)*,* both previously used by Toledo *et al*. (2013) as indicator of cellular response to hypoxia. From our selection of germline *EPAS1/*HIF-2α variants, only p.Thr766Pro alongside p.Pro531Thr positive control HIF-2α mutant was able to induce significant *CCND1* expression relative to WT (*n* = 3, *P* < 0.05) (Fig. 5B). Significant induction of *SLC2A1* was observed only in our somatic gain-of-function mutation p.Pro531Thr relative to cells transfected with WT *EPAS1* in normoxia (*n* = 3, *P* < 0.05) (Fig. 5C).

## Discussion

The role of *EPAS1*/HIF-2α in PPGL is still emerging, and the pathogenic potential of germline *EPAS1* variants is not well understood. In this study we have systematically assessed the functional effect of germline *EPAS1* variants c.581A>G (p.His194Arg), c.739C>A (p.Arg247Ser), c.1121T>A (p.Phe374Tyr), c.2296A>C (p.Thr766Pro), c.2353C>A (p.Pro785Thr) and c.2365A>G (p.Ile789Val) identified in patients with PPGL. Collectively, our data show that p.Arg247Ser, p.Phe374Tyr and p.Pro785Thr each share some features in common with the known oncogenic *EPAS1* mutation p.Pro531Thr.

We initially identified four germline *EPAS1* variants during validation of NGS testing in a cohort of (de-identified) PPGL cases. Five of six PPGL patients with these *EPAS1* variants also had germline *SDHB* mutations, although this association may have been biased by the fact that our validation cohort deliberately included a predominance of cases with known PPGL-associated mutations. When we extended *EPAS1* testing to a larger cohort (*n* = 300) including cases from Australia, Germany, Italy and Poland, we found 15 additional cases with germline *EPAS1* variants, five of whom had germline mutations in *SDH* genes. In the combined cohort, we did not find an association between *SDHx* mutations and *EPAS1* variants. Of variants present in gnomAD, only c.581A>G (p.His194Arg), c.2353C>A (p.Pro785Thr) and c.2365A>G (p.Ile789Val) were more common in PPGL patients than European non-Finnish controls. *EPAS1* variant c.739C>A (p.Arg247Ser) is novel and not present in gnomAD.

We found that *EPAS1* variants p.Arg247Ser, p.Phe374Tyr and p.Pro785Thr were stable in normoxia in diverse cellular backgrounds (HEK293, PC12 and HeLa cells). These first-line assessments suggested that residues distant from hydroxylation sites in HIF-2α could also affect its stability. We then tested whether this normoxic stability was due to failure of pVHL recognition. As expected, the known gain-of-function variant p.Pro531Thr did not interact with pVHL in normoxia; of the other variants, only our novel variant p.Arg247Ser also failed to interact with pVHL. Our structural models confirmed that *EPAS1/*HIF-2α p.Arg247 directly interacts with pVHL p.Asp143 (salt bridge) and p.Gln145 (hydrogen bond). Interestingly, it has been previously shown that the tumorigenic mutant p.Gln145His of pVHL (Gallou *et al.* 1999) failed to form a complex with HIF-2α and target it for degradation (Miller *et al.* 2005). We expect that our predicted mode of interaction is relevant during the early phase of recognition between pVHL and HIF-2α. Mutating Arg247 by a serine can disrupt the long-range electrostatic attraction of pVHL and guide pVHL to an incorrect final binding position (Shashikala *et al.* 2019). Interestingly, residue 247 was already described as being part of a transient interaction relevant to the assembly of complex HIF-2α-HIFβ/ARNT (Scheuermann *et al.* 2009, Wu *et al.* 2015).

Surprisingly, despite being stable in normoxia, p.Phe374Tyr and p.Pro785Thr variants retained interaction with p.VHL. It is possible these variants escape pVHL-mediated degradation by disrupting E3 ubiquitin ligase sites through minor torsions generated by change in amino acid residues. An alternate hypothesis is the p.Phe374Tyr variant may disrupt sumoylation at residues 394 and 497 and thereby modify ubiquitination (van Hagen *et al.* 2010). These possibilities require further investigation.

In transcriptional studies, p.Phe374Tyr and p.Pro785Thr showed similar transactivation of a HIF-2α-reporter gene compared with the positive control p.Pro531Thr. However, only p.Thr766Pro was able to induce endogenous HIF-2α-responsive *CCND1* in a manner comparable to p.Pro531Thr, while none of our HIF-2α mutants were able to significantly induce *SLC2A1* expression compared to the p.Pro531Thr control.

Taken together, these functional studies suggest that germline *EPAS1* variants p.Arg247Ser, p.Phe374Tyr and p.Pro785Thr have variable effects on protein stability, pVHL interaction and transcriptional activity that are similar to, but generally more modest, than the pathogenic somatic variant p.Pro531Thr.

Mosaic *EPAS1* variants clustered around codon 531 are unequivocally associated with the Zhuang–Pacak syndrome (Favier *et al.* 2012, Zhuang *et al.* 2012, Pacak *et al.* 2013, Yang *et al.* 2013, Jochmanova & Lazurova 2014). These same *EPAS1* mutations, presumably somatic, are found in up to 10% of sporadic PPGLs (Buffet *et al.* 2014, Crona *et al.* 2014, Comino-Méndez *et al.* 2013, Yang *et al.* 2015). Somatic/mosaic *EPAS1* mutations have also been described in PPGLs arising on a background of cyanotic congenital heart disease (Vaidya *et al.* 2018). Conversely, germline *EPAS1* variants in the oxygen-dependent degradation domain (in residues between 533 and 549) were previously identified in subjects with familial erythrocytosis type IV and rarely associated with PPGL alone (Percy *et al.* 2008). There have been three previous case reports of *EPAS1* variant c.1121T>A (p.Phe374Tyr) associated with PPGL. Lorenzo *et al.* (2013) first reported this germline variant in a 50-year-old man with EPO-dependent polycythemia and metastatic PGL, who at age 35 years had a PGL of the organ of Zuckerkandl resected; structural modeling of c.1121T>A (p.Phe374Tyr) suggested possible interference of HIF-2α interaction with elongin C as a mechanism for gain-of-function. Welander *et al.* (2014) reported the variant in a 56-year-old woman with a benign 50 mm pheochromocytoma. Taïeb *et al.* (2016) described this variant in a 63-year-old man with sporadic CNS hemangioblastoma. None of these cases were associated with a family history of pheochromocytoma or paraganglioma.

*EPAS1*/HIF-2α expression is higher in tumors associated with *VHL* or *SDHx* mutations (Qin *et al.* 2014), and recent pre-clinical data show HIF-2α synergizes with *Sdhb* deficiency to promote a metastatic cellular phenotype (Morin *et al.* 2020). It is possible therefore even mild hypermorphic activity of the germline *EPAS1*/HIF-2α variants described herein, extended over a patient’s lifetime, would also synergize with SDH deficiency (or any other pseudohypoxic stimulus) to promote PPGL development. In this regard, it would be most interesting to study PPGLs associated with germline *EPAS1* variants for a pseudohypoxic gene expression signature. Whether germline *EPAS1* variants we have described here truly modify disease expression when co-inherited with pathogenic variants in PPGL driver genes (in particular, those encoding SDH) will require further clinical studies, and in particular would benefit from large kindred studies where penetrance and disease behavior could be assessed as a function of co-inheritance of the putative modifier allele. Modifier genes for hereditary cancer syndromes have been suspected to explain variable disease penetrance, but few such genes have been identified conclusively (Pinto *et al.* 2020). At this time, we believe further evidence is needed before recommending screening for germline (as distinct from somatic) *EPAS1* variants in PPGL in routine clinical practice, nor would we recommend family screening for these variants. However, these *EPAS1* variants may yet be important in pharmacogenomics. Recent research has discovered small molecule inhibitors such as PT2385 and PT2977 that competitively bind to the PAS domain of HIF-2α and inhibit interaction with HIF-β, thereby blocking oncogenic transcriptional activities associated with pseudohypoxia (Chen *et al.* 2016, Cho *et al.* 2016, Ricketts *et al.* 2016). Genotyping should be included during clinical trials to determine whether these *EPAS1* variants affect response to HIF-2α antagonist therapies.

The strength of our study is the number of different approaches we have taken to carefully characterize each germline *EPAS1* variant, including allele frequency comparison between cases and controls, Bayesian analyses, *in silico* predictions, structural modeling and functional assessment using six different assays in transfected cells. Our study has several weaknesses: we had very limited clinical information from most subjects, and were not able to examine whether these *EPAS1* variants were associated with more aggressive disease or (other than case #3) with polycythemia; statistical comparison with gnomAD controls is limited by the very low MAF for each variant in our cases and we only used transient transfections for our functional studies, which may have missed more subtle effects on transcriptional induction by some variants and/or effects on cell proliferation. In the absence of significant changes in gene expression, we did not examine protein levels of cyclin D1 or GLUT1 following transfection of these *EPAS1* variants. Nevertheless, when taken as a whole, our results argue strongly that these germline *EPAS1* variants have at best modest effects on protein function.

In conclusion, germline *EPAS1* variants c.739C>A (p.Arg247Ser), c.1121T>A (p.Phe374Tyr) and c.2353C>A (p.Pro785Thr) share some functional features in common with the known oncogenic somatic variant p.Pro531Thr. These germline variants are unlikely to be pathogenic driver events in isolation. Whether they are co-pathogenic in combination with other events deserves further study, particularly whether they may be additive to other germline variants in *VHL/SDHx-*associated PC/PGL development.

## Supplementary Material

Supplementary Table 1: Composition of clinical cohorts according to presence of known germline driver gene mutations

Supplementary Table 2. Allele frequencies of EPAS1 variants in PPGL Cases from Australia, Germany, Italy, and Poland

Supplementary Table 3: ethnic variation in EPAS1 variants

Supplementary Fig. S1

Supplementary Fig. S2

Supplementary Fig. S3

Supplementary Fig. S4

## Declaration of interest

The authors declare that there is no conflict of interest that could be perceived as prejudicing the impartiality of the research reported.

## Funding

This work was supported by NHMRC Project 1108032 to T D, D B and R J C-B and Hillcrest Foundation (Perpetual Trustees) to D B and T D.

## Author contribution statement

T D and R C B conceived the study and wrote the manuscript. R C B, D B, and T D were responsible for curating the genetic test results. Additional oversight of the clinical cohorts was provided by G E, S R, M M, E R, A P, M P and K P. Functional studies were performed by E K with supervision by T D, D B and R C B. Structural models were built and analyzed by K B. All authors had full access to the data, and contributed to review of the manuscript.
