# Supplementary material for: Functional significance of germline EPAS1 variants
Source: Endocr Relat Cancer. 2020 Dec 7;28(2):97–109. doi: 10.1530/ERC-20-0280 (PMC7989857; doi:10.1530/ERC-20-0280)
Supplement: Supplementary Table 1: Composition of clinical cohorts according to presence of known germline driver gene mutations [file supplementary_table_1.pdf]

Supplementary Table 1: Composition of clinical cohorts according to presence of known germline driver gene mutations

	SDHB	SDHD	SDHC	SDHA	SDHAF2	VHL	FH	RET	MEN1	NONE	Total
**Australia**	34	11	4	4	0	10	1	0	0	69	133
**Germany**	3	1	2	0	1	0	0	1	1	36	45
**Poland**	20	28	2	0	0	6	0	2	0	12	70
**Italy**	18	22	3	0	0	7	0	0	0	2	52
**Total**	75	62	11	4	1	23	1	3	1	119	**300**
