# Supplementary material for: Functional significance of germline EPAS1 variants
Source: Endocr Relat Cancer. 2020 Dec 7;28(2):97–109. doi: 10.1530/ERC-20-0280 (PMC7989857; doi:10.1530/ERC-20-0280)
Supplement: Supplementary Table 2. Allele frequencies of EPAS1 variants in PPGL Cases from Australia, Germany, Italy, and Poland [file supplementary_table_2.pdf]

Supplementary Table 2. Allele frequencies of *EPAS1* variants in PPGL Cases from Australia, Germany, Italy, and Poland

Cohort/Variant	p.H194R	p.Arg247Ser	p.Ala277Val	p.Phe374Tyr	p.Gly655Arg	p.Thr766Pro	p.Pro785Thr	p.Ile789Val
**PPGL subjects**	N	N	N	N	N	N	N	N
**Australia**	1/127	1/133	0/133	3/124	1/133	6/127	1/129	1/129
**Germany**	0/42	0/42	0/42	1/44	0/44	3/42	1/42	0/42
**Italy**	0/40	0/52	1/52	0/52	0/52	2/52	0/52	0/52
**Poland**	0/70	0/70	0/70	1/70	0/70	1/70	0/70	0/70
**Total**	1/279	1/297	1/297	5/289	1/300	12/292	2/293	1/293

N: number of subjects with allelic variant/total number of subjects. MAF: minor allele frequency (number of cases with allelic variant/total number of alleles. Note that *EPAS1* coverage was not complete for some samples.
