# Supplementary material for: Functional significance of germline EPAS1 variants
Source: Endocr Relat Cancer. 2020 Dec 7;28(2):97–109. doi: 10.1530/ERC-20-0280 (PMC7989857; doi:10.1530/ERC-20-0280)
Supplement: Supplementary Table 3: ethnic variation in EPAS1 variants [file supplementary_table_3.pdf]

Supplementary Table 3: ethnic variation in *EPAS1* variants

Data from gnomAD v. 3.1

**Cohort/Variant**	**p.His194Arg**	**p.Ala277Val**	**p.Phe374Tyr**	**p.Gly655Arg**	**p.Thr766Pro**	**p.Pro785Thr**	**p.Ile789Val**
European (non-Finnish)	0	0.00001767	0.005952	0.000365	0.01651	0.0004878	0.00004396
African	0	0	0.001283	0.0002411	0.4241	0.1472	0
Latino	0.0005362	0	0.002399	0.00008474	0.02243	0.007732	0
Other	0.0001384	0.0001637	0.005812	0.0001387	0.03087	0.005949	0
South Asian	0	0.00009932	0.003103	0	0.007088	0.0001307	0
Ashenazi Jewish	0	0	0.002218	0	0.01939	0.00009645	0
East Asian	0	0	0	0.00005012	0.1307	0	0.0001087
European (Finnish)	0	0	0.003025	0.0001195	0.01935	0	0
