# Supplementary material for: Functional significance of germline EPAS1 variants
Source: Endocr Relat Cancer. 2020 Dec 7;28(2):97–109. doi: 10.1530/ERC-20-0280 (PMC7989857; doi:10.1530/ERC-20-0280)
Supplement: Supplementary Fig. S1 [file supplementary_figure_1.pdf]

Supplementary Fig. S1

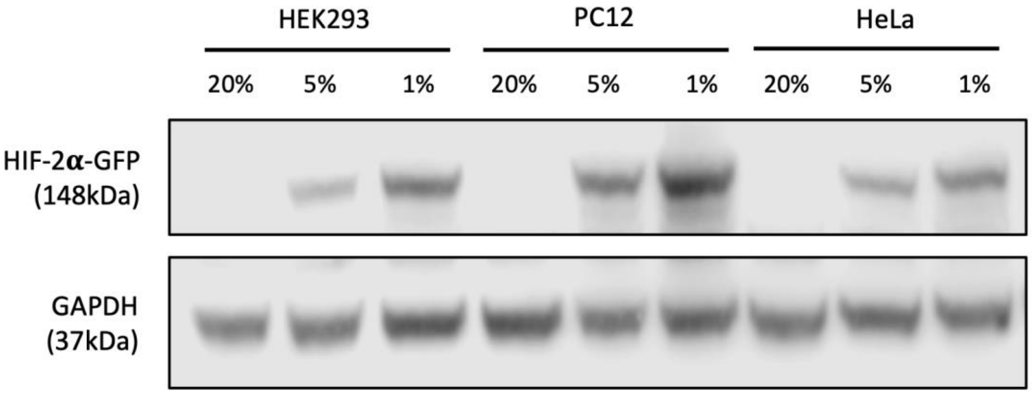

Western blotting of HEK293, PC12 and HeLa cell lysates after GFP-tagged wildtype *EPAS1* plasmid transfection over 48 hours in various oxygen levels (20%, 5%, 1% O_2). The GFP signal was observed in lysates from cells cultured in low oxygen.
