# Supplementary material for: Functional significance of germline EPAS1 variants
Source: Endocr Relat Cancer. 2020 Dec 7;28(2):97–109. doi: 10.1530/ERC-20-0280 (PMC7989857; doi:10.1530/ERC-20-0280)

Supplementary Fig. S2

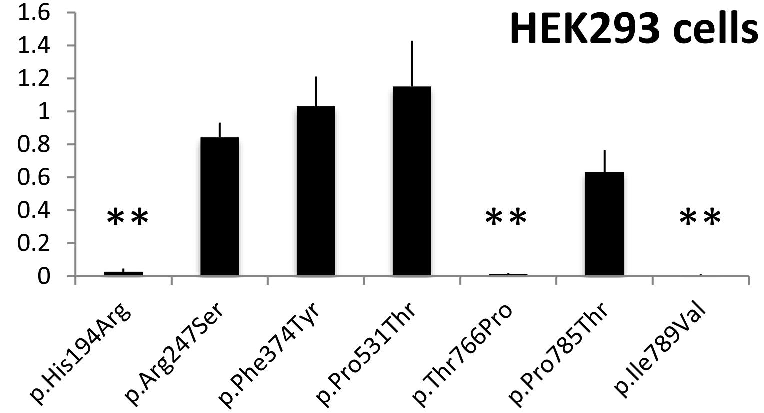

Densitometric analysis of western blots for GFP-tagged HIF-2α (WT or mutants). Data are expressed relative to WT HIF-2α under hypoxia, and are mean ± SD of three independent experiments. *p<0.05 and **p<0.005 for comparison between each mutant and positive control HIF-2α p.Pro531Thr.

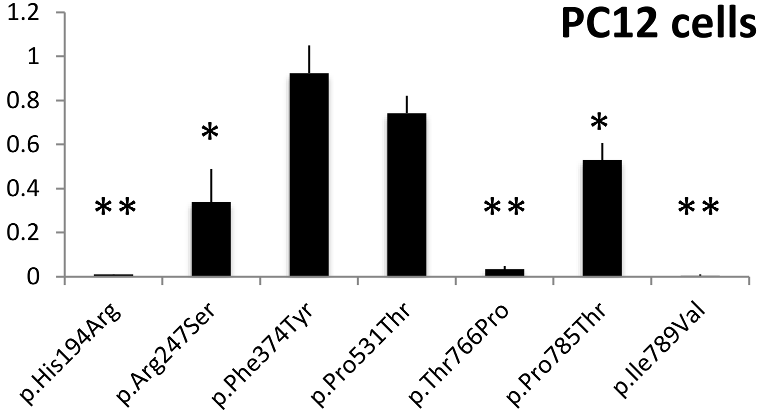

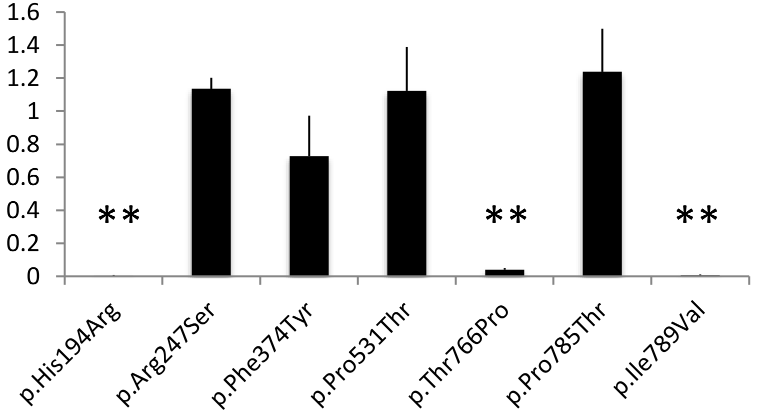

Supplement: Supplementary Fig. S2 [file supplementary_figure_2.pdf]
