# Supplementary material for: Functional significance of germline EPAS1 variants
Source: Endocr Relat Cancer. 2020 Dec 7;28(2):97–109. doi: 10.1530/ERC-20-0280 (PMC7989857; doi:10.1530/ERC-20-0280)
Supplement: Supplementary Fig. S3 [file supplementary_figure_3.pdf]

Supplementary Fig. S3

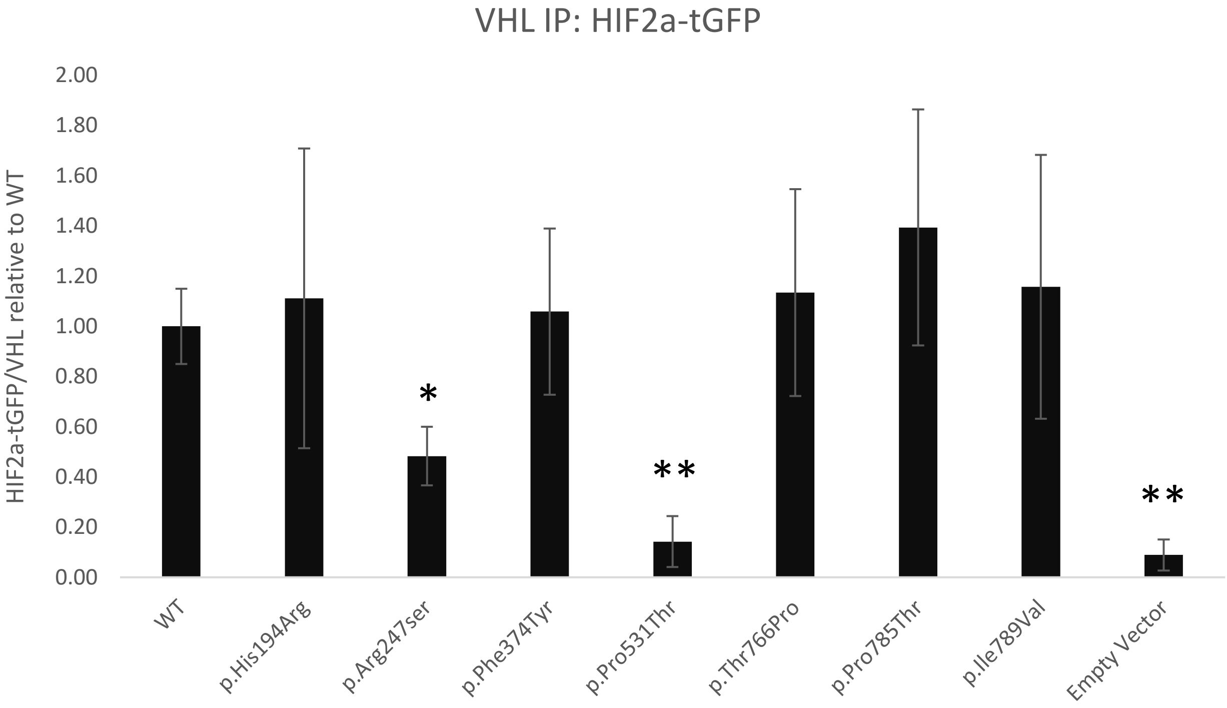

Densitometric analysis of western blots following co-immunoprecipitation of GFP-tagged HIF-2α (WT or mutants) by anti-VHL antibody, relative to HIF-2α WT in normoxia. Data are mean ± SD of three independent experiments. WT HIF-2α from cells cultured in normoxia was used as a positive control. *p<0.05 and **p<0.005 for comparison between each mutant and positive control HIF-2α WT in normoxia.
