# Supplementary material for: Functional significance of germline EPAS1 variants
Source: Endocr Relat Cancer. 2020 Dec 7;28(2):97–109. doi: 10.1530/ERC-20-0280 (PMC7989857; doi:10.1530/ERC-20-0280)

Supplementary Fig S4. Representative western blot following co-immunoprecipitation of GFP-tagged HIF-2α by anti-ARNT antibody. For positive control, HEK293 cells expressing GFP-tagged WT HIF-2α was conditioned in hypoxia (1 % O_2) over 48 hours. No obvious differences were observed between various HIF-2α mutants that were pulled down by ARNT.

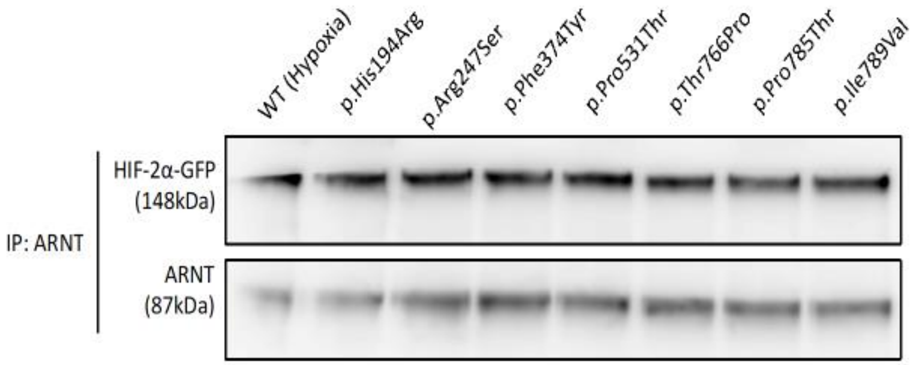

Supplement: Supplementary Fig. S4 [file supplementary_figure_4.pdf]
